# Novel small RNA spike-in oligonucleotides enable absolute normalization of small RNA-Seq data

**DOI:** 10.1038/s41598-017-06174-3

**Published:** 2017-07-19

**Authors:** Stefan Lutzmayer, Balaji Enugutti, Michael D. Nodine

**Affiliations:** Gregor Mendel Institute (GMI), Austrian Academy of Sciences, Vienna Biocenter (VBC), Dr. Bohr-Gasse 3, 1030 Vienna, Austria

## Abstract

Normalization of high-throughput small RNA sequencing (sRNA-Seq) data is required to compare sRNA levels across different samples. Commonly used relative normalization approaches can cause erroneous conclusions due to fluctuating small RNA populations between tissues. We developed a set of sRNA spike-in oligonucleotides (sRNA spike-ins) that enable absolute normalization of sRNA-Seq data across independent experiments, as well as the genome-wide estimation of sRNA:mRNA stoichiometries when used together with mRNA spike-in oligonucleotides.

## Introduction

Data from sRNA-Seq experiments are typically normalized and reported in relative terms such as reads per million genome-matching reads (RPMs)^1^. Relative normalization works well if it is assumed that the sRNA sub-populations have equal proportions across the different tissue types being profiled. However, this assumption is often invalid because sRNA populations are frequently dynamic across different tissue types and in various mutant backgrounds^2–7^. Therefore, the standard practice of comparing relatively normalized sRNA-Seq values can produce misleading results. In contrast, absolute normalization of sRNA-Seq data should enable accurate comparisons of small RNA levels in different cell types, mutant tissues or disease states on a genome-wide scale. However, previous attempts to use exogenous sRNA oligonucleotides for absolute normalization of sRNA-Seq data had variable success^8–10^ and thus have not been widely used. The varying sequencing efficiencies of specific sRNA spike-ins are most likely due to their inherent properties, such as variable secondary structures, that influence their ligation efficiency during sRNA-Seq library construction^11–13^. For example, previously an additional correction factor was required to scale individual sRNA spike-in amounts to account for the non-linear relationships between the molar amount of exogenous sRNA spike-ins added to a sample and the corresponding number of reads sequenced^10^. Here we present a novel sRNA spike-in oligonucleotide design and demonstrate how sRNA spike-ins can be used for robust absolute normalization of sRNA-Seq data across independent experiments. Moreover, we demonstrate how sRNA spike-ins can be used in combination with commercially available mRNA spike-in oligonucleotide mixes to compare values generated by sRNA-Seq and mRNA-Seq.

## Results

**Figure 1:**
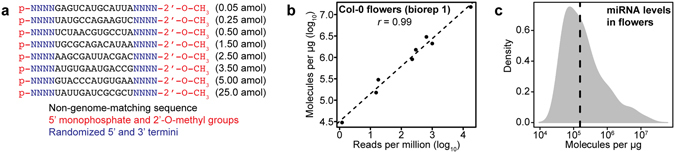
Small RNA spike-in design and use as a tool to estimate absolute small RNA levels. (**a**) Design of small RNA spike-in oligonucleotides. Key features of small RNA spike-ins are shown in different colors corresponding to the key. Molar amounts of oligonucleotides added per µg of total RNA are indicated in parentheses. (**b**) Scatter plot of relative small RNA spike-in levels (reads per million genome-matching reads) compared to absolute small RNA levels (molecules detected per µg of total RNA) in Col-0 flowers (biological replicate 1). Pearson’s *r* value is indicated, as well as a dashed line that represents a linear model derived from the plotted values. (**c**) Density plot of individual miRNA family levels in Col-0 flowers (biological replicate 1) in molecules detected per µg of total RNA. Vertical dashed line indicates the median number of molecules per miRNA family.

To generate a set of exogenous sRNA spike-ins for absolute normalization of sRNA-Seq data, we designed 21 nucleotide (nt) long RNA oligonucleotides with three main features (Fig. 1a). First, the sRNA spike-ins contained a 5′ monophosphate and 2′-O-methyl group in order to mimic endogenous plant small RNAs. The 2′-O-methyl group is common to plant small RNAs^14^, but this modification could be omitted if investigating animal miRNAs for instance. Second, the sRNA spike-ins contained a semi-random 13 nt core sequence that does not match the genome-of-interest (e.g. in this study *Arabidopsis thaliana*). Third, these semi-random 13 nt core sequences are flanked by a set of four randomized nucleotides on both the 5′ and 3′ ends. Eight sRNA spike-ins with different 13 nt core sequences were designed and mixed in specific molar ratios as shown in Fig. 1a. These were added to total RNA prior to sRNA-Seq library preparation. After sequencing, the non-genome matching 13 nt unique tags were used to quantify reads derived specifically from each sRNA spike-in. Each of these 13 nt core sequences can be represented by up to 65,536 possible 21 nt sequences. Because individual sRNA sequences have variable properties, such as secondary structure, that bias their representation in the final sRNA-Seq libraries^11–13^, the large number of diverse sequences that can be assigned to each 13 nt core sequence of the sRNA spike-in is expected to minimize cloning biases of each spike-in set as a whole. Accordingly, the sRNA spike-ins enabled the robust generation of standard curves for absolute data normalization in terms of molecules detected per µg total RNA (MPU) (Fig. 1b). Nearly perfect positive correlations (all had Pearson’s *r* values ≥ 0.99 and *P* < 7.42 × 10^−6^) were observed when plotting relative RPM levels reported by sRNA-Seq and the known absolute MPU amounts of sRNA spike-ins added to each sample (Fig. 1b and Supplementary Fig. 1a–e). As a proof of concept, the highly linear relationships between relative and absolute values were used to generate linear models to predict the number of miRNA molecules per µg of total RNA isolated from wild-type (Col-0) flowers (Fig. 1c).

**Figure 2:**
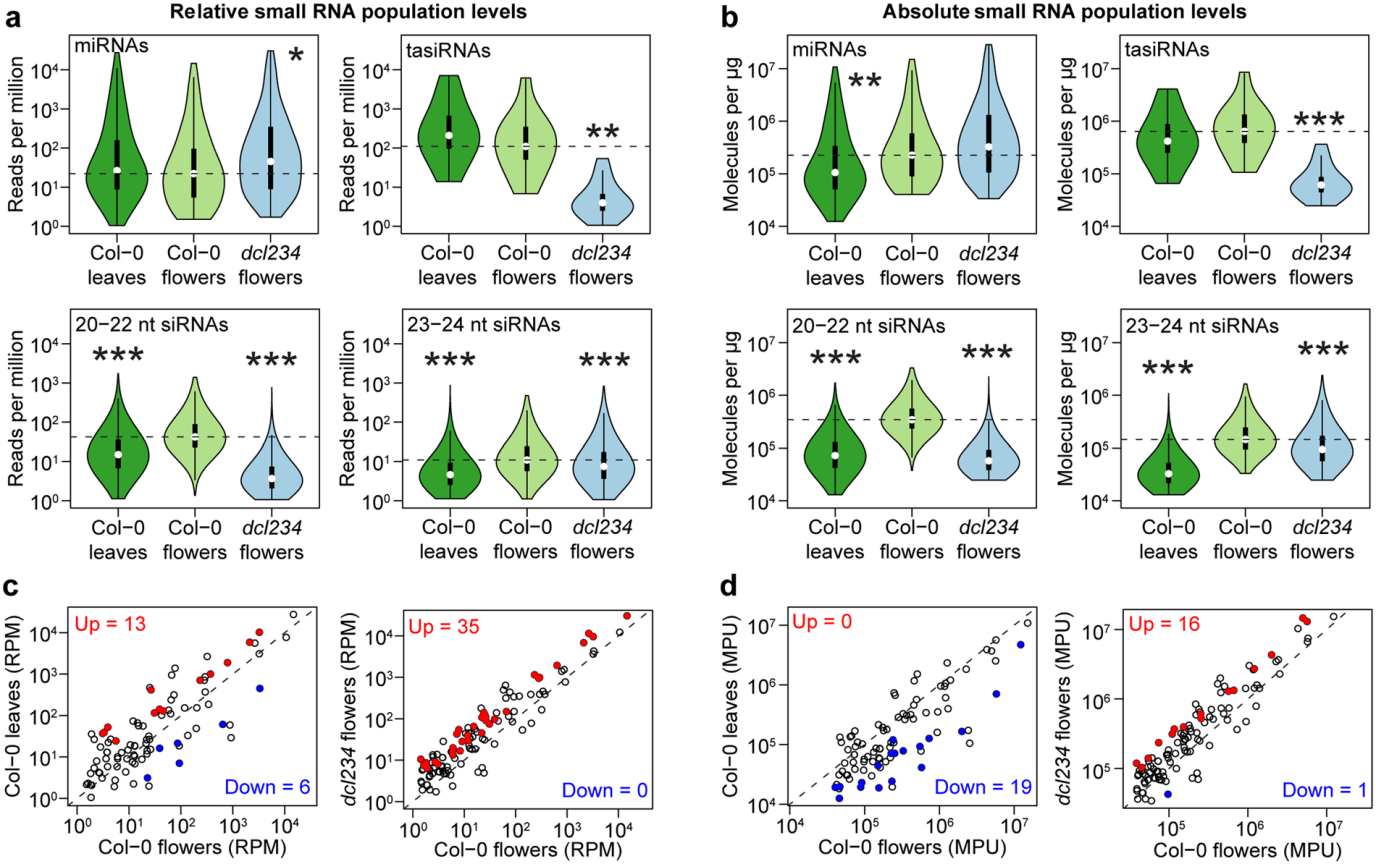
Small RNA spike-ins enable accurate comparisons of small RNA levels. (**a** and **b**) Violin plots of individual miRNAs, tasiRNAs and siRNA family levels in either relative (**a**) or absolute (**b**) units. P-values were based on two-sample Kolmogorov-Smirnov tests. *P* < 0.05, *P* < 0.01 and *P* < 0.001 are indicated by*, ** and ***, respectively. Violin plots are based on the mean among two bioreplicates for 93 miRNA, 14 tasiRNA, 6,361 20–22 nt siRNA and 5,952 23–24 nt siRNA families with ≥1 RPM in each bioreplicate. The 25^th^ and 75^th^ percentiles are indicated by the bottom and top of the vertical black bar in the violin plots, while the medians are indicated by white points within the vertical black bars. The top and bottom whiskers extend from the vertical black bar to the most extreme values within 1.5 times the interquartile range. The widths of the violins are proportional to the sample densities. (**c** and **d**) Scatter plots of the mean miRNA family levels among both bioreplicates for Col-0 flowers vs. Col-0 leaves (left) and Col-0 flowers vs. *dcl234* leaves (right) in either relative RPM (**c**) or absolute MPU (**d**) terms. Red and blue points indicate miRNA families with significantly increased and significantly decreased levels, respectively. Significantly different miRNA levels were defined as having ≥2-fold difference and a p-value < 0.05 based on a two-sample Student’s t-test.

Because the sRNA spike-ins enabled accurate data normalization (Fig. 1 and Supplementary Fig. 1a–e), we compared sRNA sub-populations in relative RPM and absolute MPU terms to determine if the two normalization procedures yield different results. Although similar analyses could be performed on any species, we used *Arabidopsis thaliana* to test the utility of the sRNA spike-ins because the *Arabidopsis thaliana* genome has well-annotated sRNAs and transcript models, different tissue types are easy to obtain and mutants deficient in major sRNA sub-populations are viable with only minor morphological effects. Plant sRNA populations consist of four main classes: 20–22 nt microRNAs (miRNAs) and trans-acting siRNAs (tasiRNAs) that tend to post-transcriptionally regulate protein-coding genes, and 20–22 nt and 23–24 nt small interfering RNAs (siRNAs) that typically arise from and silence transposons^15^. The proportions of sRNA sub-populations vary between flowers and leaves (Supplementary Fig. 2), which makes comparisons of relatively normalized leaf and flower sRNA levels prone to errors. For example, 30% and 53% of the respective Col-0 flower and Col-0 leaf sRNA populations were composed of 20–22 nt sequences (Supplementary Fig. 2a–c), but it cannot be determined whether there are absolutely more 20–22 nt sRNAs in leaves or whether this relative increase is merely due to reduced 23–24 nt siRNA levels in leaves compared to flower tissues. Using sRNA spike-ins, we compared relative and absolute normalization methods on sRNA sub-populations in Col-0 flowers and leaves. The two normalization methods produced different results. For example, whereas total miRNA levels have a significantly higher RPM in Col-0 leaves compared to Col-0 flowers (1.7-fold; *P* = 3.2 × 10^−3^; two-sample Student’s t-test), the absolute number of miRNAs are non-significantly reduced 1.5-fold in Col-0 leaves compared to Col-0 flowers (*P* = 0.13; two-sample Student’s t-test) (Supplementary Fig. 2d,e). Moreover, absolute, but not relative, normalization shows that miRNA families have significantly increased levels in Col-0 flowers compared to Col-0 leaves (Fig. 2a,b) (*P* = 4.1 × 10^−3^; two-sample Kolmogorov-Smirnov test). Therefore, relative normalization of sRNA-Seq data followed by comparisons between tissue types can lead to misleading results, which are mitigated through the use of sRNA spike-ins.

To further test how changes in sRNA populations can affect relative normalization, we generated sRNA-Seq libraries from siRNA-deficient flowers and compared these to the Col-0 flower datasets. More specifically, we generated sRNA-Seq libraries from flowers with null mutations in three genes encoding the DICER-LIKE2 (DCL2), DCL3 and DCL4 ribonucleases (i.e *dcl234* flowers). *DCL3* and *DCL4* are required for 23–24 nt siRNA and tasiRNA biogenesis, respectively^16–20^ (Supplementary Fig. 2c). Therefore, *dcl234* flowers are expected to have reduced tasiRNA and 23–24 nt siRNA levels. Comparisons of relative and absolute normalization methods on 20–24 nt siRNA levels produced similar results (Supplementary Fig. 2d,e and Fig. 2a,b). However, relative RPM levels for total tasiRNAs were not significantly reduced in *dcl234* flowers compared to Col-0 flowers; whereas, absolute MPUs for total tasiRNAs were significantly reduced (*P*_RPM_ = 0.08, *P*_MPU_ = 0.04; two-sample Student’s t-test) (Supplementary Fig. 2d,e). Both relatively and absolutely normalized tasiRNA family levels are significantly reduced in *dcl234* flowers but the decrease is more pronounced when using absolute terms (*P*_RPM_ = 1.0 × 10^−3^, *P*_MPU_ = 1.6 × 10^−4^; two-sample Kolmogorov-Smirnov test) (Fig. 2a,b). We also found that the total amount of miRNAs had both significantly higher RPMs and MPUs in *dcl234* flowers compared to Col-0 flowers (*P*_RPM_ = 0.04, *P*_MPU_ = 0.02; two-sample Student’s t-tests) (Supplementary Fig. 2d,e). Therefore, in the absence of siRNAs, miRNAs are increased as has been previously proposed^21^.

The levels of individual sRNAs are often compared across sRNA-Seq datasets from various tissue types in order to determine when and where a particular sRNA is most abundant. We compared relatively and absolutely normalized values for 93 miRNA families in Col-0 flowers versus Col-0 leaves or *dcl234* flowers to determine if the two normalization methods yield different results. Indeed, comparisons of RPM-based values suggest that 13 and 6 miRNA families have respectively increased and decreased levels in Col-0 leaves compared to Col-0 flowers (Fig. 2c). In contrast, due to the higher absolute miRNA levels in flowers (Supplementary Figure 2e and Fig. 2b), comparisons of MPU-based values indicate that no miRNA families are increased in Col-0 leaves compared to flowers, and 19 miRNA families are decreased in Col-0 leaves (Fig. 2d). Moreover, 35 miRNA families have increased levels in *dcl234* versus Col-0 flowers based on RPM values; whereas, only 16 miRNA families are increased in *dcl234* flowers compared to Col-0 flowers when using MPU values for the comparisons (Fig. 2c,d). Based on our results, absolute normalization of sRNA-Seq data from tissues with different underlying small RNA populations enables more accurate comparisons of both total and individual sRNA levels.

**Figure 3:**
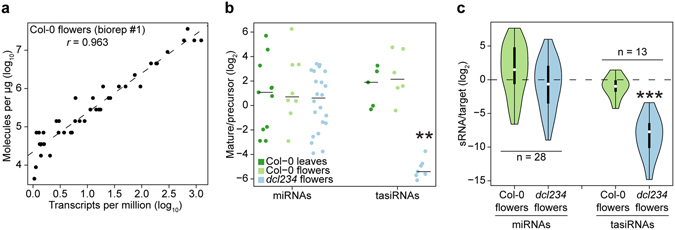
Combined use of small RNA and poly(A) RNA spike-ins allow direct comparisons of small RNA-Seq and mRNA-Seq data. (**a**) Scatter plot of relative (transcripts per million) and absolute (molecules per µg total RNA) ERCC poly(A) spike-in (LifeTech) levels. Pearson’s *r* value is indicated, as well as a dashed line that represents a linear model derived from the plotted values. (**b**) One-dimensional scatter plots of miRNA/miRNA precursor and tasiRNA/tasiRNA precursor levels in Col-0 leaves, Col-0 flowers and *dcl234* flowers. Horizontal black bars represent the median of each population. (**c**) Violin plots of miRNA/target and tasiRNA/target levels in Col-0 leaves, Col-0 flowers and *dcl234* flowers. Violin plots are as described in the Fig. 2 legend. *P* < 0.01 and *P* < 0.001 are indicated by ** and ***, respectively, and were calculated with two-sample Kolmogorov-Smirnov tests. The number (n) of miRNA:target and tasiRNA:target interactions examined is indicated.

The relationships between sRNA levels and the abundance of either their precursors or targets are important to understand miRNA biogenesis and function. Because small RNAs and their longer RNA precursors/targets require different procedures for selection and cloning into RNA-Seq libraries, it is impossible to use relative normalization approaches to estimate the stoichiometries between sRNAs and their precursors or targets. We tested whether sRNA spike-ins together with commercially available mRNA spike-ins (ERCC spike-in mixes; LifeTech) enable cross-comparisons between sRNA-Seq and mRNA-Seq datasets. More specifically, we generated mRNA-Seq datasets from the same total RNA samples used to generate the sRNA-Seq data described above, and added ERCC spike-in mixes to the total RNA prior to mRNA-Seq library construction. Strong positive correlations between relative numbers of ERCC transcripts (transcripts per kilobase million; TPM) and the known number of molecules added per µg of total RNA were observed for all six datasets having Pearson’s *r* values of at least 0.96 and *P * < 1.44 × 10^−12^ (Fig. 3a and Supplementary Fig. 3a–e). We then used these relationships to generate linear models and applied these to mRNAs with TPMs ≥ 1.0 in order to estimate mRNA MPUs genome-wide in all six datasets. This allowed us to then compare mature sRNA MPUs with those from either their precursors or targets. We found that miRNA/precursor ratios were similar in Col-0 leaves, Col-0 flowers and *dcl234* flowers with respective median miRNA/precursor values of 2.1, 1.6 and 1.5 (Fig. 3b). The ratios between tasiRNAs and their precursors were higher, but not significantly different, than miRNA/precursor ratios in Col-0 leaves and Col-0 flowers with median tasiRNA/precursor ratios of 3.7 and 4.4, respectively (Fig. 3b). In contrast, tasiRNA precursors were more abundant than mature tasiRNA levels in *dcl234* flowers with a median of 42.4-fold more precursors than tasiRNAs (Fig. 3b). This is consistent with the known requirement of *DCL4* for tasiRNA biogenesis and the corresponding increase in tasiRNA precursor levels in *dcl234* tissues^16–18, 20, 22^.

We then investigated the stoichiometries between miRNAs and their targets genome-wide. We used publically available datasets of sRNA cleavage products (i.e. degradome datasets) from Col-0 flowers to select miRNA and tasiRNA targets^23, 24^. Ratios between miRNAs and their targets were not significantly different in Col-0 and *dcl234* flowers and had median miRNA/target values of 3.9 and 0.63, respectively. In contrast, tasiRNA/target ratios were significantly different in Col-0 and *dcl234* flowers as expected (*P* = 5.0e^−16^; two-sample Kolmogorov-Smirnov test) with respective 1.5-fold and 210.8-fold more target MPUs compared to tasiRNA MPUs (Fig. 3c). Therefore, in combination with mRNA spike-ins, sRNA spike-ins allow genome-wide estimations of precursor:sRNA and sRNA:target stoichiometries.

## Discussion

Small RNA spike-ins not only serve as useful internal controls for sRNA-Seq experiments requiring only 1–2% of the alignable reads for subsequent analyses, but can also be used to normalize sRNA-Seq data from different treatments, tissue types or research groups. The absolute normalization of sRNA molecules can be essential for accurate comparisons. Moreover, sRNA spike-ins enable genome-wide estimations of precursor:sRNA and sRNA:target stoichiometries, which are important for understanding these relationships on a molecular scale. Lastly, sRNA spike-ins could be used to assess cloning biases that exist in various sRNA-Seq library generation protocols for further improvements of such methods.

## Methods

### Plant material

The *dcl234* mutants were composed of *dcl2-1*, *dcl3-1* and *dcl4-2t* alleles as previously described^17^. Plants were grown in a controlled growth chamber at 20 °C–22 °C with a 16-h light/8-h dark cycle. Col-0 leaf samples were from rosette and cauline leaves isolated from 4–6 week old plants. Unopened floral buds were either collected from the same plants as the leaf samples (Col-0 flower) or from *dcl234* plants (*dcl234* flowers) grown under identical conditions.

### sRNA spike-in design

An in-house script (https://github.com/Gregor-Mendel-Institute/sRNA-spike-ins/blob/master/methods.sRNA.spike.in.design/methods.sRNA.spike.in.design.step1.py) was used to generate a matrix consisting of the proportions of base identities for miRNA positions 5–17 (counting from the 5′ end of the miRNA) of the top 50% most highly abundant miRNAs and to semi-randomly select 1000 13 nt sequences. Two hundred and fifty-two sequences that did not perfectly align to the *Arabidopsis thaliana* Col-0 genome were considered further and all 256 possible 4-base combinations were added to both the 5′ and 3′ ends making 65,536 total sequences per set. The minimum free energies of endogenous miRNAs and all 21 nt RNAs within the 252 sets of 65,536 sequences were determined using RNAfold^25^ with the options *–T 4 –noPS* with in-house scripts (https://github.com/Gregor-Mendel-Institute/sRNA-spike-ins/blob/master/methods.sRNA.spike.in.design/methods.sRNA.spike.in.design.step2.endogenous.miRNAs.py) and (https://github.com/Gregor-Mendel-Institute/sRNA-spike-ins/blob/master/methods.sRNA.spike.in.design/methods.sRNA.spike.in.design.step2.py), respectively. Minimum free energy distributions of endogenous miRNA and sRNA spike-in secondary structures were then examined with (https://github.com/Gregor-Mendel-Institute/sRNA-spike-ins/blob/master/ methods.sRNA.spike.in.design/examine.MFE.distributions.R) and eight sets of 21 nt RNA sequences with distributions similar to annotated miRNAs were selected for synthesis and ordered from Integrated DNA Technologies (IDT) (Supplementary Figure 4). The mix ratios were formulated to span the dynamic range of annotated *Arabidopsis* miRNAs.

### RNA sequencing

Total RNA was isolated using TRIzol reagent (Thermo Fisher). The sRNA spike-in mix shown in Fig. 1a was diluted two-fold and added to 500 ng of total RNA prior to polyacrylamide gel size-selection of 18–75 nt RNAs followed by sRNA cloning using the NEBnext small RNA library prep kit for Illumina (NEB). ERCC spike-in mixes (LifeTech) were diluted 200-fold and 1 µl was added to 500 ng of total RNA. Ten ng of total RNA was used to generate mRNA-Seq libraries as described by Picelli *et al*.^26^. Samples were sequenced on an Illumina Hi-Seq 2500 sequencing machine in either single read 50 bp (sRNA-Seq) or paired-end read 50 bp (mRNA-Seq) modes. RNA-Seq datasets have been deposited into NCBI GEO (GSE98553).

### Data analysis

Adaptor sequences were removed from sRNA-Seq reads with (https://github.com/Gregor-Mendel-Institute/small-RNA-spike-ins/methods.data.analysis/small.RNA.Seq/sRNA_step1_var_qual.py) and the resulting FASTQ file was condensed and converting to FASTA format with (https://github.com/Gregor-Mendel-Institute/sRNA-spike-ins/blob/master/methods.data.analysis/small.RNA.Seq/sRNA_step2_v04.py). The adaptor-trimmed sRNA-Seq reads were then aligned to the *Arabidopsis thaliana* Col-0 genome (TAIR10) including sRNA spike-ins using the Bowtie short-read aligner^27^ with the options *–v 0 –m 100* requiring perfect matches and allowing up to 100 alignments per read (Supplementary Tables 1 and 2). Read numbers per locus were normalized for the number of times they matched the Col-0/spike-in composite genome with (https://github.com/Gregor-Mendel-Institute/sRNA-spike-ins/blob/master/ methods.data.analysis/small.RNA.Seq/bwtToAllHits_v03.py). Reads mapping to miRNA genes, tasiRNA genes, transposons and common 13 nt sequences of the sRNA spike-ins were then grouped together with (https://github.com/Gregor-Mendel-Institute/sRNA-spike-ins/blob/master/methods.data.analysis/small.RNA.Seq/make_ghcsAndlDicts_slim.py), and reads mapping to miRNA and tasiRNA genes were further organized with (https://github.com/Gregor-Mendel-Institute/sRNA-spike-ins/blob/master/ methods.data.analysis/small.RNA.Seq/miR_finder_v03.py and https://github.com/Gregor-Mendel-Institute/sRNA-spike-ins/blob/master/methods.data.analysis/small.RNA.Seq/tasiR_finder_anno_v03.py, respectively). Small RNA-Seq reads were assigned to mature miRNAs or tasiRNAs if they were 20–22 nt long and contained within ±2 nt of the sense strand of the miRNA or tasiRNA according to annotations in miRBase21^28^ and Allen *et al*.^29^, respectively. Values for individual miRNAs or tasiRNAs belonging to common families were then added together to obtain the total amount of reads for individual families. Small RNA reads which were either 20–22 nt or 23–24 nt long and overlapped either strand of TAIR10 annotated transposons (i.e. transposable elements and transposable element genes) were grouped according to the transposon that they mapped to, and are referred to as siRNAs. miRNAs, tasiRNAs, 20–22 nt siRNAs and 23–24 nt siRNAs were grouped together based on the above criteria using (https://github.com/Gregor-Mendel-Institute/sRNA-spike-ins/blob/master/methods.data.analysis/small.RNA.Seq/getMirsSirsAndTas_v02.py and https://github.com/Gregor-Mendel-Institute/sRNA-spike-ins/blob/master/methods.data.analysis/small.RNA.Seq/getMirsSirsAndTas_v03.py), and the levels of miRNAs and tasiRNAs coming from each annotated precursor was quantified with (https://github.com/Gregor-Mendel-Institute/sRNA-spike-ins/blob/master/ methods.data.analysis/small.RNA.Seq/getMirAndTasPrecursorVals.py). Python scripts were used to quantify the RPM levels for 18–30 base reads as well as the base frequencies at each position with (https://github.com/Gregor-Mendel-Institute/sRNA-spike-ins/blob/master/methods.data.analysis/small.RNA.Seq/getLengthVals.py).

Paired-end mRNA-Seq reads were aligned to the *Arabidopsis thaliana* Col-0 genome (TAIR10) and ERCC spike-ins using RSEM^30^ with default parameters. ERCC spike-in and Araport11^31^ transcript models were then quantified and recorded in (https://github.com/Gregor-Mendel-Institute/sRNA-spike-ins/blob/master/ methods.data.analysis/master_genes_Araport.txt).

### Statistical analysis

Statistical analyses were performed and graphics were generated with R^32^. Biological replicates were included for each sRNA-Seq and mRNA-Seq dataset, and were defined as independently acquired pooled collections of tissues from separate plants. R scripts used to generate all the figures used in this study are publicly available at https://github.com/Gregor-Mendel-Institute/sRNA-spike-ins/.

## Electronic supplementary material

Supplementary Information
