# Supplementary material for: Novel small RNA spike-in oligonucleotides enable absolute normalization of small RNA-Seq data
Source: Sci Rep. 2017 Jul 19;7:5913. doi: 10.1038/s41598-017-06174-3 (PMC5517642; doi:10.1038/s41598-017-06174-3)
Supplement: Supplementary file 1 — Supplementary Information [file 41598_2017_6174_MOESM1_ESM.pdf]

Supplementary Information for:

Novel small RNA spike-in oligonucleotides enable absolute normalization of small RNA-Seq data

Stefan Lutzmayer, Balaji Enugutti and Michael D. Nodine

Supplementary figures

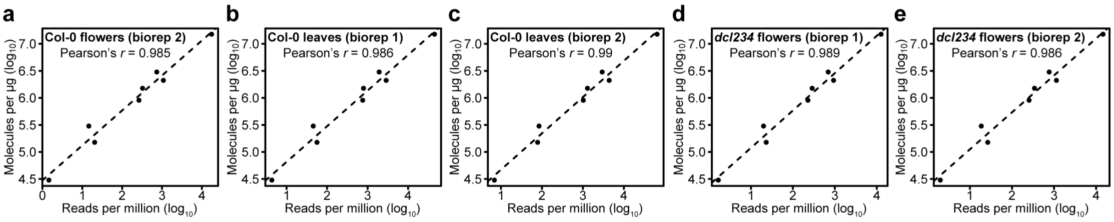

Supplementary Figure 1 | Linear relationships between the number of reads sequenced and amount of sRNA spike-in oligos added to each sample. Scatter plot of relative small RNA spike-in levels (reads per million genome-matching reads) compared to absolute small RNA levels (molecules per µg of total RNA) in Col-0 flowers (biological replicate 2) (**a**), Col-0 leaves (biological replicates 1 and 2) (**b** and **c**) and *dcl234* flowers (biological replicates 1 and 2) (**d** and **e**). Pearson's *r* values are indicated, as well as dashed lines that represent linear models derived from the plotted values.

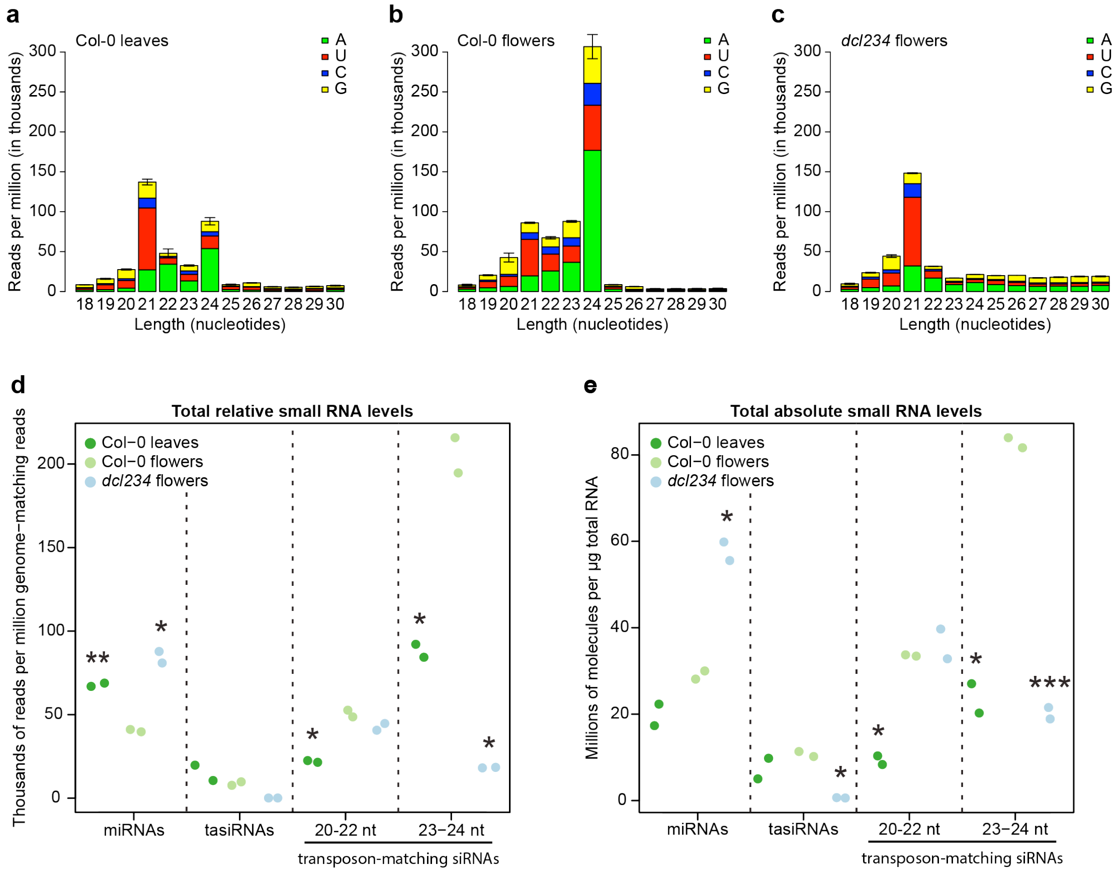

Supplementary Figure 2 | Length distributions and quantification of small RNA populations. (**a** - **c**) Stacked bar plots of RPMs for different size classes of sRNAs in Col-0 leaves (**a**), Col-0 flowers (**b**) and *dcl234* flowers (**c**). Colors indicate the proportion of sRNA-Seqs that begin with the indicated nucleotides. Error bars indicate the standard error of the mean based on two biological replicates. (**d** and **e**) One-dimensional scatter plots of miRNA, tasiRNA, 20-22 nt siRNA and 23-24 nt siRNA levels in relative units (thousands of reads per million genome-matching reads) (**d**) or absolute units (millions of molecules per µg total RNA) (**e**) in Col-0 leaves, Col-0 flowers and *dcl234* flowers. Two-sample Student's t-tests were used to determine p-values in (**d** and **e**), and $P < 0.05$, $P < 0.01$ and $P < 0.001$ are indicated by *, ** and ***, respectively.

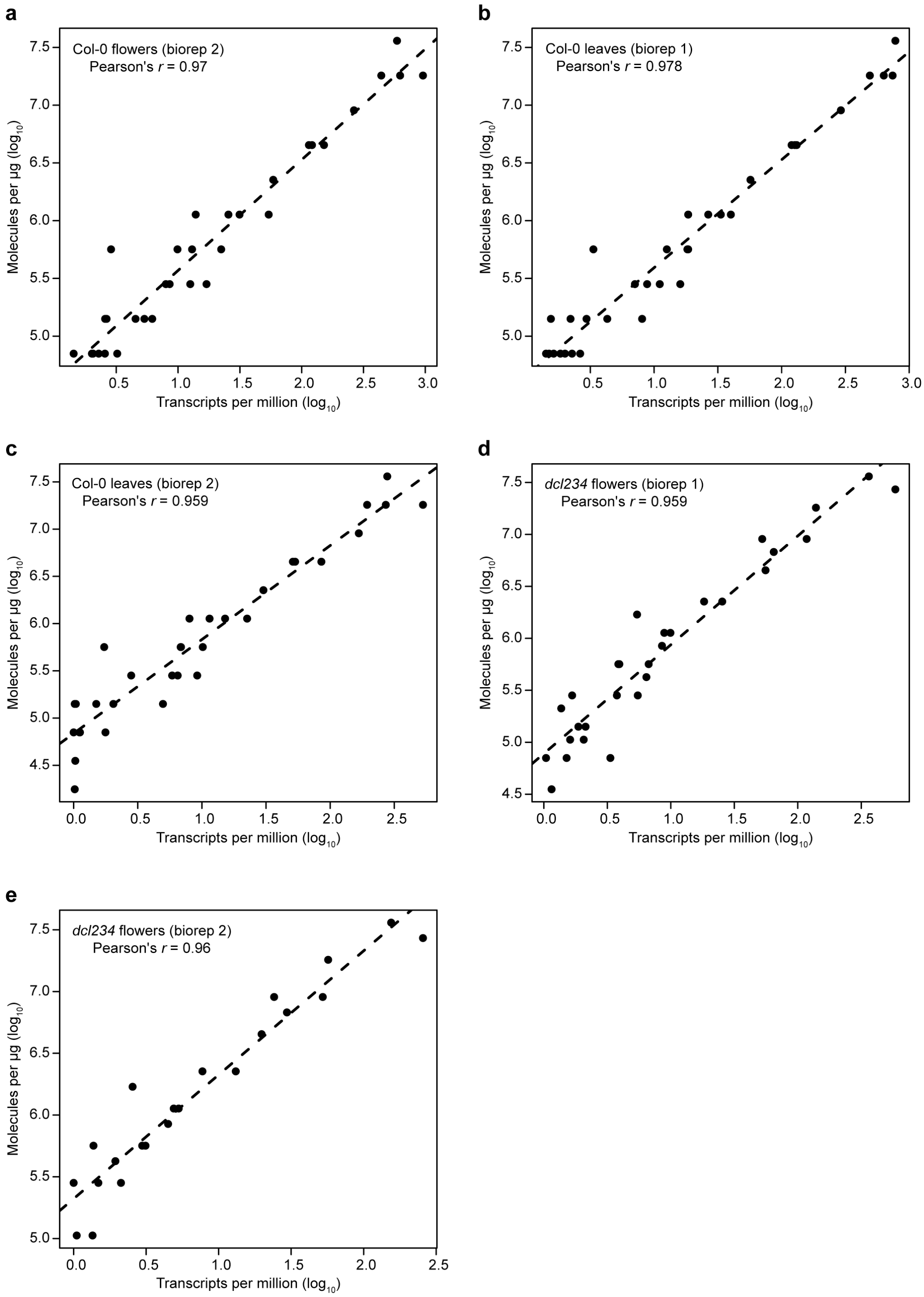

Supplementary Figure 3 | Standard curves used to estimate the number of mRNA molecules per µg of total RNA. Scatter plots of relative (transcripts per million) and absolute (molecules per µg total RNA) ERCC poly(A) spike-in levels for mRNA-Seq libraries generated from wild-type (Col-0) flowers (biological replicate 1) (**a**), Col-0 leaves (biological replicates 1 and 2) (**b** and **c**) and *dcl234* flowers (biological replicates 1 and 2) (**d** and **e**). Pearson's *r* values are indicated, as well as dashed lines that represent linear models derived from the plotted values.

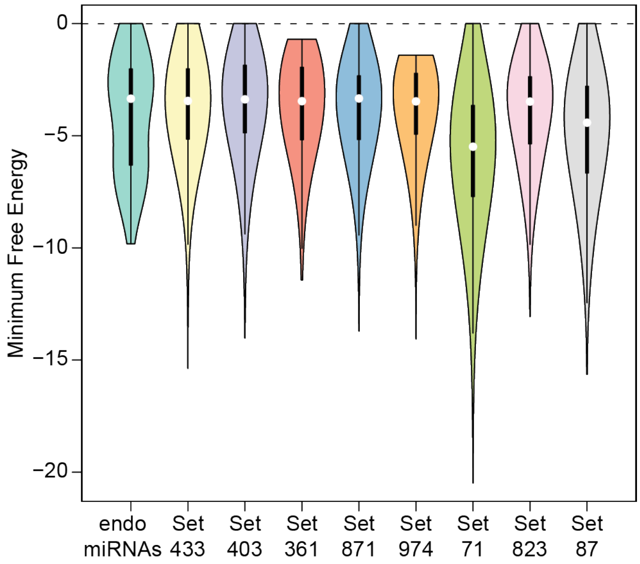

Supplementary Figure 4 | Minimum free energy distributions of endogenous miRNAs and sRNA spike-in secondary structures. Minimum free energy distributions for the secondary structures of the top 50% expressed endogenous miRNAs (endo miRNAs) and eight sets of semi-randomly generated oligonucleotide sequences (Sets) that were chosen as sRNA spike-ins.

Supplementary tables

Supplementary Table 1 | Statistics for sRNA-Seq libraries

Library[a]	Col-0 leaf #1	Col-0 leaf #2	Col-0 flowers #1	Col-0 flowers #2	*dcl234* flowers #1	*dcl234* flowers #2
Genome-matching reads[b]	8,786,408	4,062,090	13,243,396	13,755,078	10,164,548	8,590,559
sRNA spike-in matching reads	451,446	303,113	261,989	274,476	150,279	147,114
% sRNA spike-in reads	5.14%	7.46%	1.98%	1.99%	1.48%	1.71%

[a] #1 and #2 indicate biological replicates 1 and 2, respectively
[b] genome-matching reads also include the number of reads that map to either ERCC or sRNA spike-ins

Supplementary Table 2 | Statistics for mRNA-Seq libraries

**Library**[a]	**Col-0 leaf #1**	**Col-0 leaf #2**	**Col-0 flowers #1**	**Col-0 flowers #2**	***dcl234* flowers #1**	***dcl234* flowers #2**
Genome-matching reads[b]	9,204,152	9,156,745	1,4357,321	11,836,228	12,193,753	14,294,965
mRNA spike-in matching reads	19,072	8,199	29,000	21,057	10,354	5,391
% mRNA spike-in reads	0.21%	0.09%	0.20%	0.18%	0.08%	0.04%

[a] #1 and #2 indicate biological replicates 1 and 2, respectively

[b] genome-matching reads also include the number of reads that map to ERCC spike-ins
